# Transforming growth factor β-induced epithelial-to-mesenchymal signature predicts metastasis-free survival in non-small cell lung cancer

**DOI:** 10.18632/oncotarget.26574

**Published:** 2019-01-25

**Authors:** Edna Gordian, Eric A. Welsh, Nicholas Gimbrone, Erin M. Siegel, David Shibata, Ben C. Creelan, William Douglas Cress, Steven A. Eschrich, Eric B. Haura, Teresita Muñoz-Antonia

**Affiliations:** ^1^ Tumor Biology Program, H. Lee Moffitt Cancer Center and Research Institute, Tampa, FL, USA; ^2^ Cancer Informatics Core, H. Lee Moffitt Cancer Center and Research Institute, Tampa, FL, USA; ^3^ Molecular Oncology Program, H. Lee Moffitt Cancer Center and Research Institute, Tampa, FL, USA; ^4^ Cancer Epidemiology Program, H. Lee Moffitt Cancer Center and Research Institute, Tampa, FL, USA; ^5^ Department of Surgery, University of Tennessee Health Science Center, Memphis, TN, USA; ^6^ Department of Thoracic Oncology, H. Lee Moffitt Cancer Center and Research Institute, Tampa, FL, USA; ^7^ Department of Biostatistics and Bioinformatics, H. Lee Moffitt Cancer Center and Research Institute, Tampa, FL, USA

**Keywords:** non-small cell lung cancer, epithelial-to-mesenchymal transition, EMT, metastasis, colon cancer

## Abstract

Transforming growth factor beta (TGFβ) plays a key role in regulating epithelial-to-mesenchymal transition (EMT). A gene expression signature (*TGFβ-EMT*) associated with TGFβ-induced EMT activities was developed using human Non-Small Cell Lung Carcinoma (NSCLC) cells treated with TGFβ-1 and subjected to Affymetrix microarray analysis. The final 105-probeset *TGFβ-EMT* signature covers 77 genes, and a NanoString assay utilized a subset of 60 of these genes (TGFβ-EMTN signature). We found that the *TGFβ-EMT* and *TGFβ-EMT_N_* gene signatures predicted overall survival (OS) and metastasis-free survival (MFS). The TGFβ-EMT signature was validated as prognostic of 5-year MFS in 3 cohorts: a 133 NSCLC tumor dataset (*P* = 0.0002), a NanoString assays of RNA isolated from formalin-fixed paraffin-embedded samples from these same tumors (*P* = 0.0015), and a previously published NSCLC MFS dataset (*P* = 0.0015). The separation between high and low metastasis signature scores was higher at 3 years (ΔMFS *TGFβ-EMT* = −28.6%; ΔMFS *TGFβ-EMT_N_* = −25.2%) than at 5 years (ΔMFS *TGFβ-EMT* = −18.6%; ΔMFS *TGFβ-EMT_N_* = −11.8%). In addition, the *TGFβ-EMT* signature correlated with whether the cancer had already metastasized or not at time of surgery in a colon cancer cohort. The results show that the *TGFβ-EMT* signature successfully discriminated lung cancer cell lines capable of undergoing EMT in response to TGFβ-1 and predicts MFS in lung adenocarcinomas. Thus, the *TGFβ-EMT* signature has the potential to be developed as a clinically relevant predictive biomarker, for example to identify those patients with resected early stage lung cancer who may benefit from adjuvant therapy.

## INTRODUCTION

Early-stage non-small cell lung carcinoma (NSCLC) recurrences are attributable to metastatic disease undetected at the time of resection [1]. In the first step of metastasis, tumor cells dissociate and migrate from the primary tumors as a consequence of epithelial-to-mesenchymal transition (EMT), a process involving the induction of transcription factors through multiple signaling pathways that, together, change cell adhesion and migration properties [2]. Signaling pathways involved in the induction of EMT include Transforming Growth Factor β (TGFβ), Wnt-β-catenin, Bone Morphogenetic Protein, Notch, Hedgehog, and some receptor tyrosine kinases [2]. Specifically, TGFβ has been shown to induce EMT in NSCLC, which may lead to an increased potential to invade and disseminate [3].

TGFβ is a cytokine involved in numerous cellular processes, including growth, proliferation, adhesion, migration, and apoptosis [4]. TGFβ signal transduction begins with ligand binding to the TGFβ type II receptor, followed by recruitment of the type I receptor and formation of a hetero-oligomeric complex of TGFβ-1, TGFβ type II receptor, and TGFβ type I receptor [5]. After complex formation, the constitutively autophosphorylated TGFβ type II receptor phosphorylates the TGFβ type I receptor, initiating a phosphorylation cascade of downstream cytoplasmic substrates, including the SMAD proteins, with subsequent activation of target genes [4]. The crosstalk between the TGFβ pathway and many other signal transduction pathways results in modification of the original TGFβ signal through non-canonical pathways, and is used to explain the multiple effects of TGFβ [6–8]. In normal epithelial cells, TGFβ inhibits cell proliferation and induces apoptosis, thereby acting as a tumor suppressor; however, TGFβ also acts as a tumor promoter, as it plays a role at many levels of carcinogenesis. These include epithelial/mesenchymal differentiation via SMADs and PI3K-AKT, angiogenesis via activating vascular endothelial growth factor and metalloproteases [9], and evasion of immune suppression by inhibiting the growth of many hematopoietic cell lines and by impairing T-cell activation [10, 11].

Correlations between a TGFβ-induced gene expression signature and clinical outcomes have been described [12–14]. In some of these studies, cell lines were used for initial identification of a specific TGFβ-response gene signature, and these signatures have been correlated with overall survival (OS) using publicly available databases [15]. For example, Coulouarn and colleagues compared the TGFβ response of primary hepatocytes isolated from a TGFβ receptor knockout mouse model (unresponsive to TGFβ) and from wild type mice (responsive to TGFβ), and were able to identify early and late TGFβ signatures that predict different clinical outcomes in human hepatocellular carcinomas [13]. When they used these two signatures to query archived gene expression profiles of lung adenocarcinomas, they found the same results for patients with hepatocellular carcinoma (patients with a late TGFβ signature had significantly shortened median survival time compared to patients who had an early TGFβ signature). Also, using breast carcinoma cell lines, Padua and colleagues used the gene expression profile of response to TGFβ to define a signature specific for lung metastasis (as opposed to bone metastasis) and identified *ANGPTL4* (angiopoietin-like 4) as one of the genes induced by TGFβ involved in this mechanism [14]. In recent years, the emphasis has been on the development of TGFβ-induced EMT signatures as a tool for the prognosis and treatment of metastatic cancers (see Table 1 in Foroutan *et al.* [15]). Interestingly, there is very little overlap among the genes in the different signatures, likely due to either the number or type of cell lines used, time of TGFβ exposure, or different normalization methods. Using these signatures, Foroutan *et al.* used a bioinformatics approach to generate a signature, which identified tumors in The Cancer Genome Atlas (TCGA) with evidence of TGFβ-induced EMT. Among these tumors, tumors with high scores showed significantly lower overall survival (OS) rates than those with low scores.

**Table 1:** Characteristics and TGFβ response of NSCLC cell lines

Tumor Type	Adenocarcinoma Cell Line
*EGFR* wild type	A549, Calu-6, H23, H292, H322, H358, H441, H522, H1395, H1437, H1648, H1944, H2122, H2347
*KRAS* wild type	H292, H322, H522, H1395, H1437, H1648, H2347
*KRAS* mutant	A549, Calu-6, H23, H358, H441, H1944, H2122
Primary lesions	A549, Calu-6, H23, H322, H522, H358, H1395, H2347
Metastatic lesions	H292, H441, H1437, H1648, H1944, H2122
**Response to TGFβ**	
Growth Inhibition	A549, H23, H441, H1944
Smad2-p	A549, Calu-6, H23, H292, H322, H358, H441, H1395, H1437, H1944, H2122, H2347
Decreased E-cadherin 1	A549, H358, H1944
Increased Migration	A549, H358, H1944

There are several robust prognostic gene expression signatures in NSCLC that predict poor outcomes [1, 16–19]; however, numerous reviews have pointed out the complexities of moving these from the discovery stage into clinical application [20–23]. Herein, we describe the development of a gene expression signature associated with TGFβ's tumor-promoting EMT activities (*TGFβ-EMT* signature) that works in a NanoString format in formalin-fixed paraffin embedded (FFPE) tissues. We demonstrate, through bioinformatics analysis, that this signature can identify lung cancer cell lines capable of undergoing EMT in response to TGFβ-1, and is transferable to human tumors. Most importantly, we demonstrate that the *TGFβ-EMT* signature, in both the microarray and NanoString format, can predict not only overall survival (OS), but also metastasis-free survival (MFS) in patients with NSCLC.

## RESULTS

### Gene expression in NSCLC after TGFβ-induced EMT

NSCLC cell lines can undergo TGFβ-induced EMT, implicating EMT in the development of metastasis from the lung [24, 25]; however, different NSCLC cell lines vary in their responses to TGFβ and in their capacity to undergo TGFβ-induced EMT [26] *in vitro*. Therefore, in this study, we used fourteen NSCLC cell lines and characterized them in terms of their response to TGFβ-1, via measurements of: growth, morphology, migration, SMAD-2 phosphorylation, transcriptional activity, and expression of EMT markers (E-cadherin 1, vimentin, SNAIL). Table 1 summarizes these characteristics. All cell lines in this study were WT *EGFR*; 7 were WT *KRAS* (H292, H322, H522, H1395, H1437, H1648, and H2347) and 4 were WT *TP53* (A549, H292, H1394, and H1944). Cells were categorized as EMT if they responded to TGFβ-1 (Supplementary Figure 1) and if they had EMT-associated changes after treatment with TGFβ-1. Calu-6 was excluded from the final analysis, as it is constitutively mesenchymal [26].

**Figure 1:**
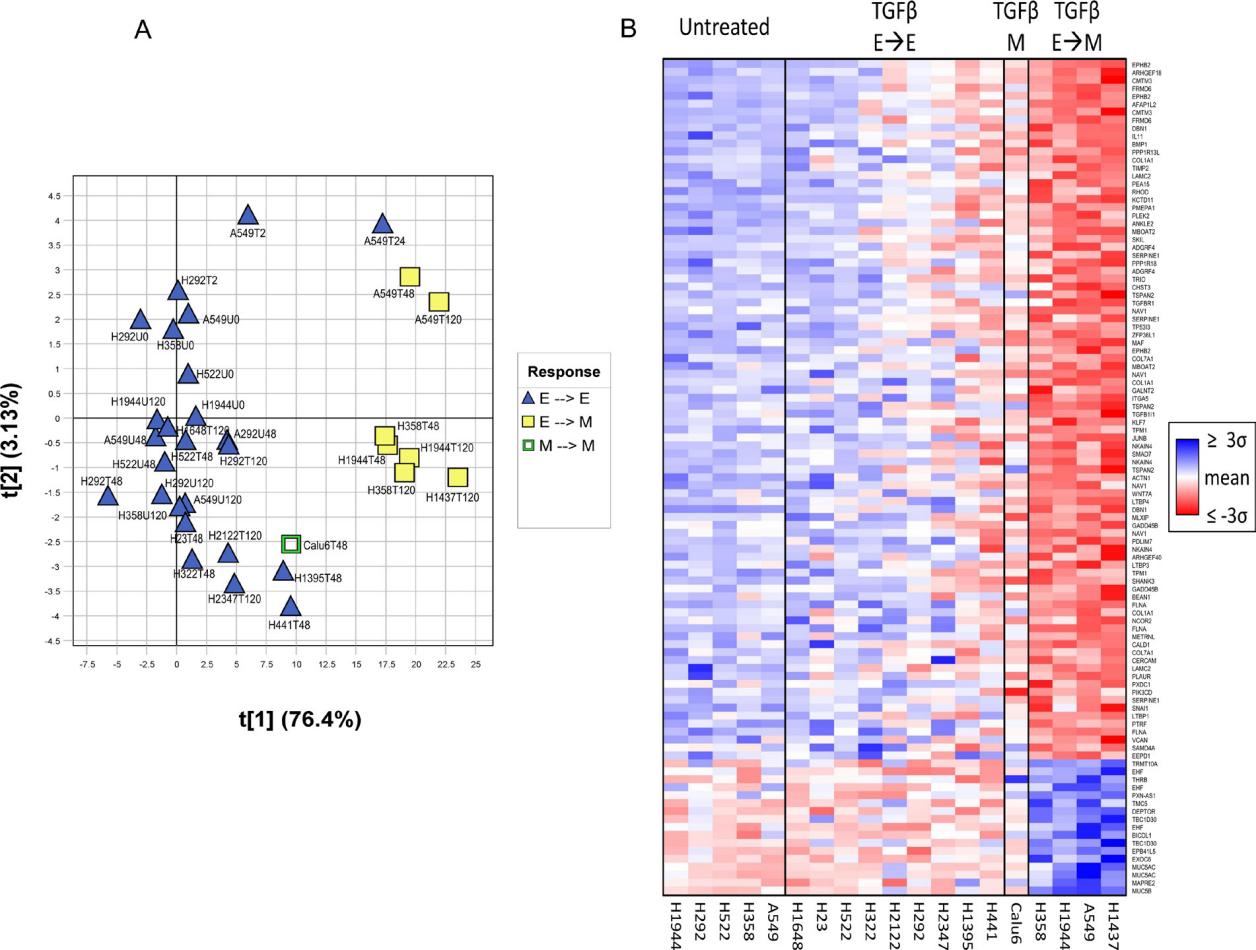
Separation of cell lines based on EMT capacity using the *TGF*β*-EMT* signature (**A**) Principal component analysis (PCA) performed on the *TGF*β*-EMT* signature, separating cell lines that underwent TGFβ-induced EMT (H358, A549, H1437, H1944) versus those that did not (H23, H292, H322, H441, H522, H1395, H1648, H2122, and H2347). Samples from cells either untreated (U) or treated with TGFβ (T) were collected at different time points (0, 2, 24, 28, 72 and 120 hours). Sample scores for the first two principal components (t[1], t[2]) are plotted on the X- and Y- axes. Percent variation captured is given in parentheses for each principal component. TGFβ-treated A549 exhibits mesenchymal gene expression at 24 hours, even though it had yet to exhibit a mesenchymal phenotype. (**B**) Heatmap of TGFβ-treated cell line experiment. Individual probesets within the signature exhibit stronger signal as samples become more mesenchymal. Samples were sorted left-to-right by treatment + phenotype group (untreated, treated with no epithelial-to-mesenchymal transition, treated with constitutively mesenchymal, treated with epithelial-to-mesenchymal transition), and then sorted within groups by the first principal component of the signature applied to all samples. Probesets were sorted vertically by their corresponding PCA loadings and colored by the mean-centered unit-variance scaled values used in the PCA. For each cell line, only the latest time point is shown for clarity (48 hour: Calu6, H23, H322, H441, H522, H1395; 120 hour: A549, H292, H358, H1437, H1648, H1944, H2122, H2347).

Gene expression changes in these cells after TGFβ treatment were determined using Affymetrix U133 Plus 2.0 microarrays. Principal component analysis (PCA) of the resulting data cleanly separated TGFβ-treated cell lines that underwent EMT when exposed to TGFβ-1 from cell lines that did not undergo EMT (Figure 1A). As part of the validation process, some cell lines were treated for longer time periods to ensure that lack of EMT response was not due to differences in doubling time (T120 time points in Figure 1A). To identify changes in gene expression associated with a TGFβ-induced EMT phenotype, cell lines that responded to TGFβ and underwent TGFβ-induced EMT (H358, A549, H1437, and H1944) were compared with those that did not (H23, H292, H322, H441, H522, H1395, H2122, and H2347). Changes in gene expression in cell lines undergoing EMT were validated by qRT-PCR on cDNA obtained from TGFβ treated and untreated NSCLC cell lines. qRT-PCR with a panel of 5 genes (*SERPINE, SMAD7, SNAI1, MUC5AC, PLAUR*) confirmed the cell specificity and direction of changes identified in the microarray analysis (Supplementary Figure 2).

### *TGFβ-EMT* signature

**Figure 2:**
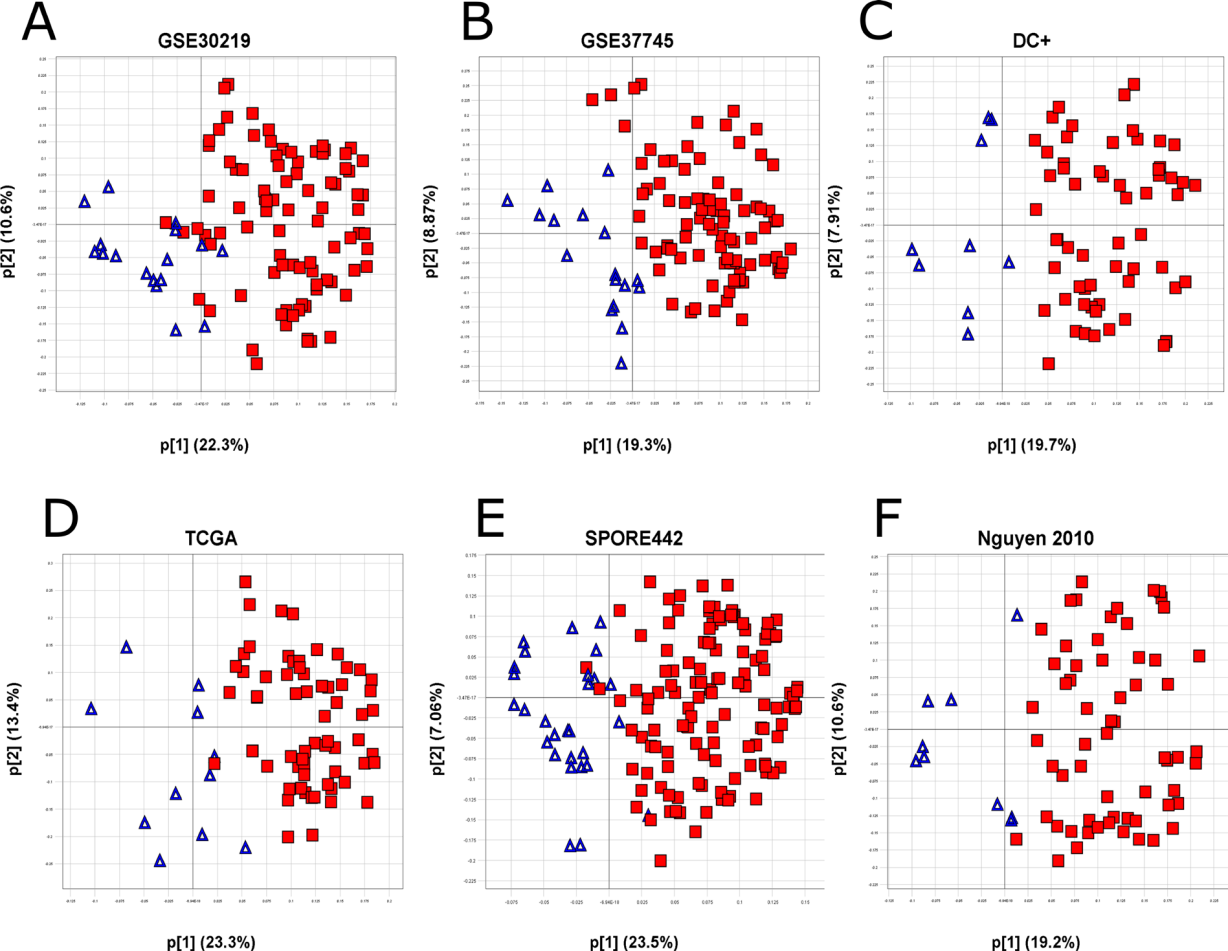
Transferability of the TGFβ-EMT signature to tumors The TGFβ-EMT signature was applied to 6 different datasets (GEO datasets GSE30219 (**A**), GSE37745 (**B**), and the Director's Challenge Plus (**C**), TCGA (**D**), the Schabath 442 cohort (**E**), and the combined cohorts from Nguyen *et al.* (**F**)). Variable loadings for the first two principal components (p[1], p[2]) are plotted on the X- and Y- axes. Percent variation captured is given in parentheses for each principal component, with large p[1]/p[2] loading ratios indicating strong signature biology [48]. The loadings for the first two principal components are plotted for each dataset and colored by the sign of the probeset in the original cell line experiment (red: up-regulated; blue: down-regulated).

The initial 1,201 differentially expressed probesets were reduced to an intermediary 135-probeset signature, consisting of the probesets exhibiting the strongest biological separation, along with a few additional less-strong genes of EMT-related biological interest. Because cell lines are a greatly simplified system compared with human lung adenocarcinoma tissue samples, the genes identified in the cell line experiment may behave differently in human tumors. Using these 135 probesets, a PCA of human lung adenocarcinomas from several external public datasets was used to identify genes that did not translate well into human tumors (see Materials and Methods), resulting in a final translated 105-probeset signature covering 77 genes, the *TGFβ-EMT* signature (Figure 1B and Table 2). A good separation of the genes is observed with loadings of these final 105 probesets in the translational datasets described in Materials and Methods, along with loadings from TCGA [27], the Schabath 442 cohort [28], and the combined cohorts from Nguyen *et al.* [29]. As shown in Figure 2, the behavior of the signature in lung tumor cohorts was similar to that shown in the cell line experiment, with positively (red) and negatively (blue) differentially expressed probesets generally clustering opposite each other, indicating good transferability between cell lines and lung tumors.

**Table 2:** Genes included in the *TGFβ-EMT* signature

Symbol		
*ACTN1*	*IL11*	*PPP1R13L*
*ADGRF4*	*ITGA5*	*PPP1R18*
*AFAP1L2*	*JUNB*	*PTRF*
*ANKLE2*	*KCTD11*	*PXDC1*
*ARHGEF18*	*KLF7*	PXN-AS1^*^
*ARHGEF40*	*LAMC2*	*RHOD*
*BEAN1*	*LTBP1*	*SAMD4A*
BICDL1^*^	*LTBP3*	*SERPINE1*
*BMP1*	*LTBP4*	*SHANK3*
*CALD1*	*MAF*	*SKIL*
*CERCAM*	MAPRE2^*^	*SMAD7*
*CHST3*	*MBOAT2*	*SNAI1*
*CMTM3*	*METRNL*	TBC1D30^*^
*COL1A1*	*MLXIP*	*TGFB1I1*
*COL7A1*	MUC5AC^*^	*TGFBR1*
*DBN1*	MUC5B^*^	THRB^*^
DEPTOR^*^	*NAV1*	*TIMP2*
*EEPD1*	*NCOR2*	TMC5^*^
EHF^*^	*NKAIN4*	*TP53I3*
EPB41L5^*^	*PDLIM7*	*TPM1*
*EPHB2*	*PEA15*	*TRIO*
EXOC6^*^	*PIK3CD*	TRMT10A^*^
*FLNA*	*PLAUR*	*TSPAN2*
*FRMD6*	*PLEK2*	*VCAN*
*GADD45B*	*PMEPA1*	*WNT7A*
*GALNT2*		*ZFP36L1*

^*^ = Negative PCA coefficient

### *TGFβ-EMT* signature and lung cancer driver mutations

**Figure 3:**
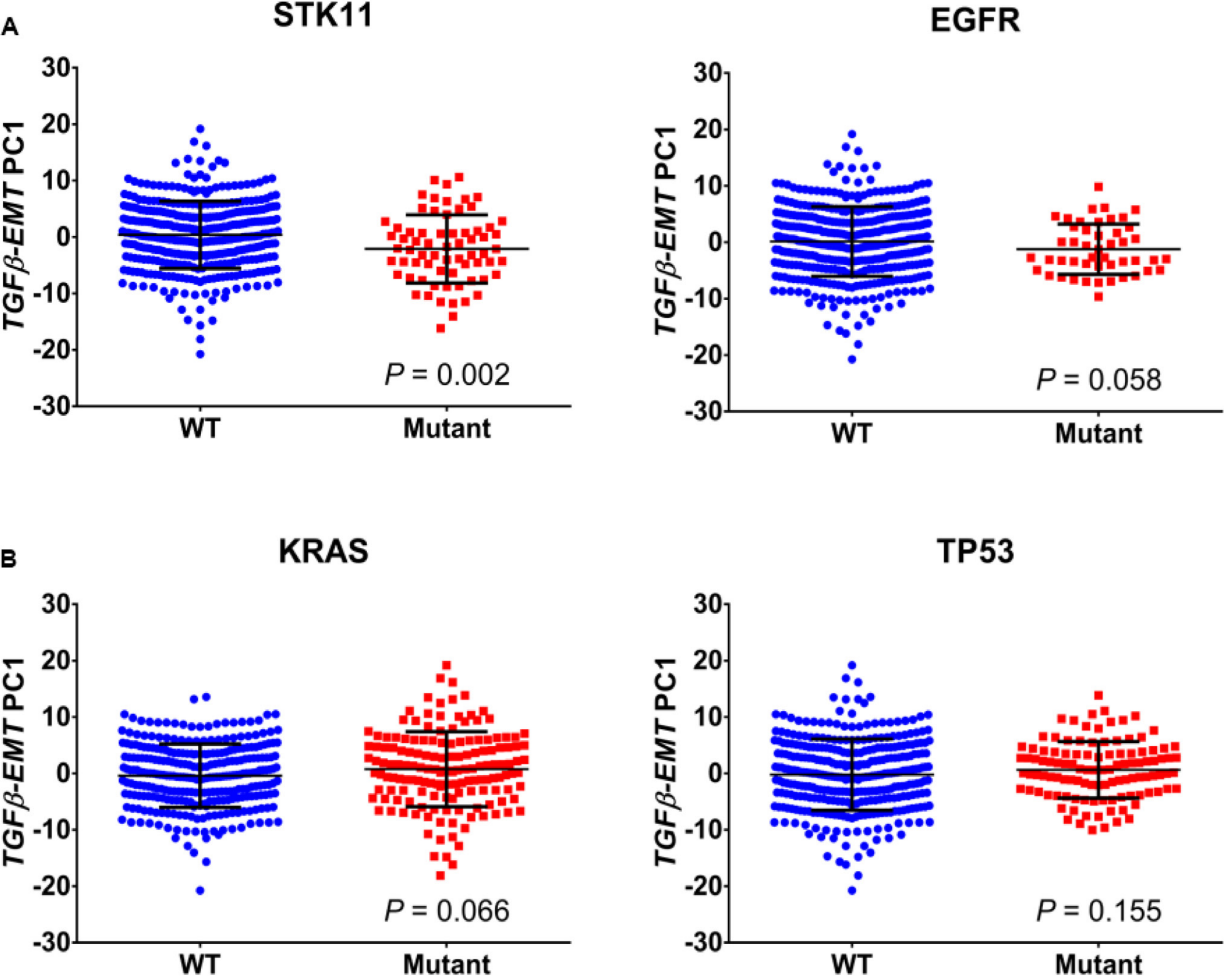
Association of *TGFβ-EMT* signature with mutations (**A**) Scores from the first principal component of the *TGFβ-EMT* signature applied to the Schabath 442 cohort are plotted for both wild-type (WT) and mutant, for four common lung adenocarcinoma mutations. Signature scores are generally lower in STK11-mutant tumors (*P* = 0.002) compared with WT. The other three mutations do not differ significantly from WT. (**B**) Genes from the *TGFβ-EMT* signature were used to cluster *STK11* mutant patients in the TCGA database into cohorts that represent a high and low signature phenotype. These patients were then analyzed by Fisher's exact test to determine if there were mutations associated with the TGFβ phenotype that drive the *STK11* mutant population. Kelch-like ECH-associated protein 1 (*KEAP1*), hepatocyte growth factor (HGF) ZNF831 (Zinc Finger Protein 831).

Using the Schabath 442 cohort, we looked for correlations between the *TGFβ-EMT* signature and driver mutations in NSCLC. As shown in Figure 3, no association was found with mutation status of *EGFR* (*P* = 0.058), *TP53* (*P* = 0.155), or *KRAS* (*P* = 0.066); however, a slight association was found with *STK11* mutations (*P* = 0.002). A possibility is that, in *STK11* mutants, the environment is altered, allowing cells to metastasize. Interestingly, TCGA patients with an *STK11* mutation have significantly lower levels of *TGF*β*-1* gene expression level (Supplementary Figure 3). Therefore, we next investigated which mutations were associated with TGFβ biology and whether these mutations could be related to EMT. For this analysis, the TCGA database was used to identify mutations associated with the *TGFβ-EMT* signature score in NSCLC patients with *STK11* mutations. K-means clustering was used to separate patients into low and high signature scores. A Fisher's exact test of each gene revealed an enrichment of *KEAP1* (Kelch-like ECH-associated protein 1) and *HGF* (Hepatocyte growth factor) mutations in patients with a low *TGFβ-EMT* signature score and *ZNF831* mutations in patients with a high *TGFβ-EMT* signature score (Figure 3B).

### *TGFβ-EMT* signature and metastasis-free survival

**Figure 4:**
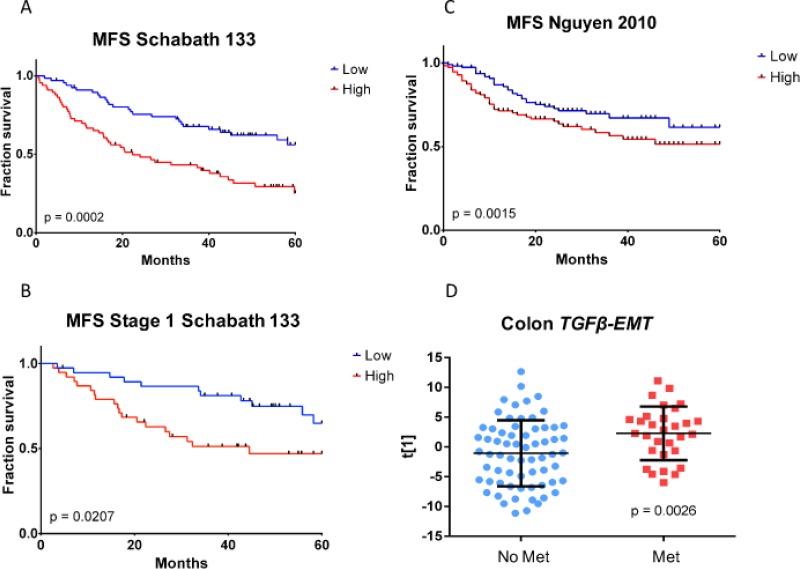
Kaplan–Meier metastasis-free survival analysis of *TGFβ-EMT* signature in fresh frozen samples (**A**) Samples with high *TGFβ-EMT* signature score (red curve) exhibit significantly worse metastasis-free survival than samples with low *TGFβ-EMT* signature score (blue curve). (**B**) Magnitude of the difference in fraction survival between high (red curve) and low (blue curve) groups is similar within the early-stage subset of the cohort. (**C**) Kaplan–Meier metastasis-free survival analysis of the *TGFβ-EMT* signature in external fresh frozen Nguyen cohort [29]. Samples with high *TGFβ-EMT* signature score (red curve) exhibited significantly worse metastasis-free survival, although the magnitude of the difference between high (red curve) and low (blue curve) groups was smaller than in the cohort used for the TMA. (**D**) Differences in *TGFβ-EMT* signature score distributions within resected primary colon tumors. Primary tumors from patients with pre-existing metastatic disease at time of surgery (red circles) exhibit higher *TGFβ-EMT* signature scores than those from patients whose tumors had not metastasized (blue circles).

Overall survival (time from date of surgery to last contact or death) and MFS (survival in which metastasis and death were both counted as events) were estimated for a 136-sample FFPE tumor subset of the Schabath 442 microarray dataset (Table 3). Three patients had a pre-existing metastasis at time of surgery, and thus were excluded from the MFS analyses (OS: *n* = 136, MFS: *n* = 133). Our results (Table 4) showed that the *TGFβ-EMT* signature not only predicted 5-year overall survival in general (*P* = 5.7 × 10^−5^), but, more specifically, also predicted 5-year metastasis-free survival (*P* = 1.6×10^−4^ (Figure 4A)). Furthermore, if only the samples from Stage I patients were evaluated, the differences between the high and low metastasis samples remained significant (Figure 4B; *P* = 0.0207). Significant differences between high and low metastasis (*P* = 0.0015) were observed when we examined the *TGFβ-EMT* signature in a combined cohort of the two NSCLC datasets from Nguyen *et al.* [29] (Figure 4C). It is interesting to note that in this cohort of 231 tumors, there were twice as many patients who developed metastases, suggesting that this cohort was more aggressive. In addition, the *TGF*β*-EMT* signature was applied to a cohort of 96 colon tumors [30], and the first principal component was used to assign *TGFβ-EMT* signature strength to each tumor, which was then used for a two-group comparison, using a two-sided *t-*test, between patients who had metastases at the time of tumor resection and those who did not (Figure 4D). This analysis demonstrated a statistically significant association between the *TGF*β*-EMT* signature and presence of metastases in colon cancer patients (*P* = 0.0026), demonstrating that targeting this conserved pathway results in a signature that works in other cancer types.

**Table 3:** Characteristics of patients included in the 133-sample subset of the Schabath 442 fresh frozen tumor microarray dataset

Patient characteristic	
Mean age, years (range)	69.2 (50–87)
Sex, No. (%)	
Male	63 (47)
Female	70 (53)
Race, No. (%)	
White	128 (96)
Black	3 (2)
Other	2 (2)
Smoking history, No. (%)	
Ever-smoker	110 (83)
Never-smoker	7 (5)
Not available	16 (12)
Disease stage, No. (%)	
I	4 (3)
IA	50 (38)
IB	22 (17)
IIA	5 (4)
IIB	16 (12)
IIIA	16 (12)
IIIB	10 (8)
IV	10 (8)
Recurrence, No. (%)	40 (30)
Metastasis, No. (%)	33 (25)

**Table 4:** Association of immunohistochemistry staining with survival in formalin fixed paraffin-embedded samples

	Overall survival		Metastasis-free survival	
	3 year	5 year	3 year	5 year
***TGFβ-EMT***	**4.62 × 10^−4^**	**5.69 × 10^−5^**	**1.37 × 10^−3^**	**1.59 × 10^−4^**
***TGFβ-EMT_N_***	**5.82 × 10^−4^**	**8.78 × 10^−4^**	**5.91 × 10^−4^**	**1.49 × 10^−3^**
**A2A**	0.736	0.619	0.890	0.559
**BTLA**	0.486	0.745	0.439	0.865
**CDH1**	0.112	**0.018**	0.091	**0.023**
**CTLA4**	0.371	0.100	0.424	0.195
**INOS**	0.481	0.973	0.593	0.697
**LAG3**	0.375	0.397	**0.050**	**0.021**
**PDL-1**	0.792	0.839	0.599	0.415
**PLAUR**	0.346	0.189	0.877	0.812
**SMAD7**	0.409	0.572	0.161	0.242
**TIM3**	0.219	0.316	0.388	0.532

Kaplan–Meier log-rank test *P*-values are reported for immunohistochemistry staining of 10 genes. MFS ratios at 3- and 5- yrs. represent the proportion of patients that have not suffered an event (metastasis or death). The *TGFβ-EMT_N_* signature was significantly associated with both OS and MFS at both 3 and 5 years. Positive E-cadherin 1 (CDH1) staining is significantly associated with OS (*P* = 0.018) and MFS (*P* = 0.023) only at 5 years. Interestingly, lack of LAG3 signal is significantly associated with MFS 5 years (*P* = 0.021), and borderline significant at 3 years (*P* = 0.050).

### *TGFβ-EMT* signature NanoString assay

**Figure 5:**
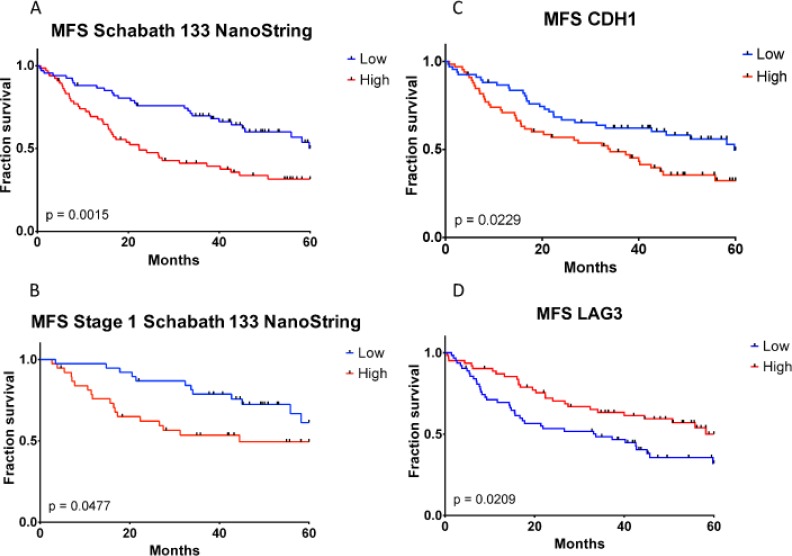
Kaplan–Meier metastasis-free survival analysis of *TGFβ-EMT* and *TGFβ-EMT_N_* signatures and CDH1 and LAG3 immunohistochemistry staining in formalin-fixed paraffin-embedded samples *TGFβ-EMT_N_ high*, CDH1 *high*, and LAG3 *high* are colored as red; *TGFβ-EMT_N_ low*, CDH1 *low*, and LAG3 *low* are colored as blue. (**A**) Samples with high *TGFβ-EMT* signature score exhibit significantly worse metastasis-free survival. (**B**) Magnitude of the difference between high and low groups is similar within the early-stage subset of the cohort. Immunohistochemistry staining for CDH1 (**C**) and LAG3 (**D**) are not associated as strongly with MFS as the *TGFβ-EMT* or *TGFβ-EMT_N_* signatures, as they exhibit higher *P*-values and lesser separation between high and low curves than in (**A**) and (**B**).

Given the potential of predicting development of metastasis, the *TGFβ-EMT* signature was adapted into a NanoString assay, which is amenable to use with FFPE samples. The subset of 60 genes included in the *TGFβ-EMT* signature NanoString Assay (*TGFβ-EMT_N_* signature) is shown in Supplementary Table 1. The *TGFβ-EMT_N_* signature was used with RNA isolated from FFPE blocks of a 133-sample subset of the Schabath 442 cohort, and a statistically significant difference in MFS was observed (Figure 5A; *P* = 0.0015; ΔMFS 3 yr. = −28.6%; ΔMFS 5 yr. = −18.6%). As with the *TGFβ-EMT* signature, if only samples from Stage I patients were evaluated, the differences between the high and low groups remained significant in the *TGFβ-EMT_N_* signature (Figure 5B; *P* = 0.0477; ΔMFS 3 yr. = −25.2%; ΔMFS 5 yr. = −11.8%).

Several molecules have been proposed as biomarkers for lung cancer progression, predictors of response to therapy or response to immunotherapy [31–33]. To examine whether the *TGFβ-EMT* signature or the NanoString assay-adapted *TGFβ-EMT_N_* signature would provide an advantage over these biomarkers, expression levels of EMT and immune biomarkers were correlated with OS and MFS in a lung adenocarcinoma Tissue Microarray (TMA) [34]. This lung cancer TMA contained 150 lung adenocarcinomas with 133 cases, overlapping with the Schabath 442 cohort, and having recurrence and metastasis data available that were used to determine the association between the *TGFβ-EMT* signature and MFS. We stained the lung adenocarcinoma TMA with antibodies against an EMT marker (E-cadherin 1 (CDH1), molecules included in the signature (SMAD7, PLAUR), and immune checkpoint markers. As shown in Table 4, the strongest positive correlations with MFS were that of the *TGFβ-EMT* signature microarray (3-yr: *P* = 1.4 × 10^−3^, 5-yr: *P* = 1.6 × 10^−4^) and *TGFβ-EMT_N_* signature NanoString (3-yr: *P* = 5.9×10^−4^, 5-yr: *P* = 1.5 × 10^−3^) assays. The correlation with staining for either the EMT biomarker E-cadherin 1 (CDH1, *P* = 0.023; Supplementary Figure 4 and Supplementary Table 2) or the lack of expression of lymphocyte activation gene-3 (LAG3, *P* = 0.021) were also statistically significant to a lesser degree, and the CDH1 high and LAG3 high (red curves) show lower MFS than the CDH1 low and LAG3 low (blue curves) (Figures 5C and 5D). Therefore, we propose that the *TGFβ-EMT_N_* signature, in a NanoString format that uses FFPE samples, can serve as a better predictor for those patients who go on to develop metastases.

## DISCUSSION

Most patients who die from lung cancer die from metastatic disease. Current therapeutic regimens have been ineffective in the cure of metastatic cancer; thus, an urgent need remains to predict which patients will go on to develop metastases. Gene signatures represent gene expression changes consistently observed after perturbation of a biological process under a limited set of experimental conditions. The behavior of genes within a signature derived from cell line experiments is expected to differ to some extent in tumors compared with cell lines. This could be due to many factors such as the immortalization of cell lines in the laboratory, the simplification of a complex multi-tissue/organ biological system into a single cell type growing on a plate, and the possible presence of additional gene expression drivers not probed in the initial cell line experiments that may confound the expression of genes within the signature. For a signature to be translatable into the more complex, but more clinically relevant, context of a tumor population, genes within a signature must be further selected for similar behavior within a tumor population.

Bioinformatics analyses, which compared cell lines that undergo TGFβ-induced EMT with those that do not, identified 1,201 probesets potentially involved in TGFβ-induced EMT. Pathway analysis of these genes with GeneGO Metacore identified cytoskeleton remodeling/cell adhesion and EMT as the main pathways affected in TGFβ-induced EMT (data not shown). Other pathways identified included cell proliferation, DNA damage, and immune response pathways. TGFβ-1 stimulation induces mesenchymal cells to secrete collagens such as collagen 7A1 (*COL7A1*), decrease protease production, and increase the secretion of protease inhibitors such as TIMPs and SERPINE1 [35], all of which are differentially expressed in cells undergoing TGFβ-induced EMT. As expected, the probesets differentially expressed include molecules from other pathways through which TGFβ signals, such as *PIK3CD*. Other genes identified (e.g., *IL11, LTBP1/2, SERPINE*) have been previously reported to be regulated by TGFβ in other microarray studies, suggesting that these TGFβ-regulated genes are not tumor type-specific, and are generally regulated by TGFβ [13, 14, 36]. Interestingly, several molecules involved in the negative regulation of the TGFβ pathway are up-regulated in cell lines that undergo EMT (e.g., *SMAD7, SMURF1*), whereas members of the TGFβ canonical SMAD pathway (e.g., *SMAD2*) are down-regulated. This suggests that one of the responses in cells that undergo TGFβ-induced EMT is to turn on this negative feedback loop to desensitize the cells to the action of TGFβ treatment, resulting in a signaling switch from the canonical pathway to other pathways. *KEAP1* interacts with nuclear factor (erythroid-derived 2)-like 2 (Nerf2), and the KEAP1/Nerf2 pathway is considered a master regulator of oxidative stress responses. Recent studies have also shown an inhibitory role of *KEAP1* in the TGFβ-1 stimulated response pathway [37]. Interestingly, in cells undergoing TGFβ-induced EMT changes (e.g., increases in fibronectin 1 and collagen 1A1), *Nerf2* activity was decreased. In this system, knockdown of *KEAP1* results in repression of TGFβ signaling (SMAD transcriptional activity) and an increase in *SMAD7* expression, both of which are part of the *TGFβ-EMT* signature. *HGF* (Hepatocyte growth factor) is a gene that has been shown to play an antagonistic role to TGFβ signaling [38]. Additionally, it has been shown to induce EMT in NSCLC, further linking its function to the TGFβ pathway [39].

The final derivation of the *TGFβ-EMT* signature was created by applying the cell line derived signature to several patient-derived tumor specimen cohorts, then identifying and removing genes that exhibit opposite behavior between cell lines and human tumors; thus, minimizing variations due to *in vitro* manipulations. This resulted in a signature that predicts both overall survival and metastasis free survival. Most of the TGFβ-induced EMT signatures described in the literature, report an association between a high TGFβ signature score and OS [15], but in some studies it is the abrogation of TGFβ signaling that correlates with OS [12], and very few [29] report an association with MFS. Most importantly, the *TGFβ-EMT_N_* signature (NanoString format), also predicts MFS using FFPE samples commonly collected in the community.

Immune evasion is required for tumor progression, and recent reports in the literature have pointed toward a connection between EMT and response to immune checkpoints, which balance self-tolerance and tissue destruction and are expressed by many tumors to inhibit anti-tumor immune responses. Recently, Mak and associates [40] found a correlation between a lung cancer EMT signature that predicts resistance to tyrosine kinase inhibitors in lung cancer and immune checkpoint inhibitors. Furthermore, Lou and colleagues examined the TCGA (The Cancer Genome Atlas), PROSPECT (Profiling of Resistance patterns and Oncogenic Signaling Pathways in Evaluation of Cancers of the Thorax), and BATTLE-1 (Biomarker-integrated Approaches of Targeted Therapy for Lung Cancer Elimination) datasets and found that adenocarcinomas displaying a mesenchymal phenotype are associated with a distinct tumor microenvironment that includes elevated levels of PD1, PDL1, PDL2, TIM3, BTLA, and CTLA4 [41]. This association has also been seen in breast cancer, where an association between an EMT signature and PDL1 up-regulation was reported [42]. These reports suggest a role for EMT markers as predictors of response to immunotherapy. In a lung adenocarcinoma TMA, protein expression of immune checkpoint molecules (A2A, BTLA, CTLA4, INOS, TIM3, and PDL1) did not correlate with MFS or overall survival. Therefore, other possibilities, such as the differences in mutation burden in tumors with high TGFβ signature scores [15], should be examined to understand the relationship between EMT and immune response

There is some overlap between different EMT signatures, suggesting some common EMT-related changes in gene expression; however, from our work and the work of others, it is clear that different EMT drivers result in different genes differentially expressed in different tissues. For instance, Nguyen and associates [29] analyzed six pathway-specific gene expression signatures (*TGFβ, KRAS, TCF4, SRC, E2F3*, and *MYC*) in cohorts of lung adenocarcinomas and primary breast tumors for which the MFS status was available, and identified signatures that can predict breast cancer recurrence (*TGFβ* signature) and lung cancer recurrence (*TCF4* and *MYC* signatures). More recently, Fouran and colleagues, using bioinformatics, derived a TGFβ-induced EMT signature, and concluded that, “there is significant overlap between our signature and other previously described signatures, suggesting some common EMT traits that should be included in assays used to identify patients who would most likely metastasize” [15]. Since Stage I lung cancer patients as a group do not benefit from adjuvant therapy, and 40–50% are not cured with surgery alone, a biomarker such as the *TGFβ-EMT* signature that is predictive of relapse with the development of metastatic disease has the potential to identify patients who may possibly benefit from adjuvant therapy. In addition, identifying which patients will survive metastasis free will spare them the time and expense of therapy.

## MATERIALS AND METHODS

### Cell culture

The following human lung adenocarcinoma cell lines were obtained from the American Type Culture Collection (Manassas, VA): NCI- H23, H292, H322, H358, H441, H522, H1395, H1437, H1648, H1944, H2122, H2347, CALU-6, and A549. NSCLC cell lines were cultured in RPMI-1640 medium (Thermo Fisher Scientific, Waltham, MA) supplemented with 10% fetal bovine serum (Atlanta Biologicals, Inc., Lawrenceville, GA), 100 U/mL penicillin, 100 μg/mL streptomycin, and 1 mM glutamine. The cell lines were maintained in a humid incubator at 37°C and 5% CO_2_.

### Microarray

Lung adenocarcinoma cell lines were treated with TGFβ-1 (5 ng/mL). Recombinant human TGFβ-1 protein was purchased from R&D Systems (Minneapolis, MN) and reconstituted in 4-mM HCL and 1-mg/mL bovine serum albumin solution. RNA was collected at various times (0, 24, 48, and 120 hours), processed, converted to cDNA, amplified, biotin-labeled, and hybridized to Affymetrix U133 Plus 2.0 microarrays (Thermo Fisher Scientific) by Moffitt Cancer Center's Molecular Genomics Core.

### Microarray analysis

Microarrays were normalized against the median sample using IRON [43]. To reduce differences in gene expression due to basal differences between cell lines, log_2_ ratios were calculated for each sample versus the average of the untreated controls for its respective cell line.

The samples were then classified as *Untreated*, *no-EMT* (TGFβ treated, no EMT), *EMT* (TGFβ treated, EMT), and *TGFβ* (*EMT* + *no-EMT* groupings), and compared as follows: *EMT* versus *no-EMT*, *EMT* versus other, *EMT* versus *Untreated*, *no-EMT* versus *EMT*, *no-EMT* versus other, *no-EMT* versus *Untreated*, and *TGFβ* versus *Untreated*. Classification as “Other” indicates samples other than the current samples of interest. For each two-group comparison, probesets were determined to be differentially expressed if the following conditions were met: the average within the experimental group and the difference between the averages of the two groups agree in sign and the absolute value of the difference is ≥ ~0.585 (1.5-fold).

Probesets were then categorized as *EMT*-related if significant in all three *EMT* groupings and in opposite direction to *no EMT* groupings (if present), *no-EMT*-related if significant in all three *no-EMT* groupings and in opposite direction to the *EMT* groupings (if present), or *TGFβ*-related if all three versus *Untreated* groupings were of the same sign. Only a single probeset, 240185_at (anti-sense to TMCO1), was classified as both *EMT*-related and *no EMT*-related, indicating strongly opposite expression behavior between the two phenotypes; this probeset was thus removed from further analysis. This initial filtering resulted in 1,201 EMT-related probesets (corresponding to > 900 genes).

These initial 1,201 probesets were further pruned into a 135-probeset signature (representing 100 genes) by keeping those most strongly associated with TGFβ-induced EMT (128 probesets), as well as an additional 7 probesets that, while less strong, are of general EMT-related biological interest (DDR1, LTBP1, PDGFB, SMURF1, SNAI1, TGFBR1). Identification of the strongest probesets was determined by first requiring complete separation between the two groupings being compared in at least one of three EMT grouping comparisons. The worst (lowest magnitude) of the three comparisons must then pass a 2-fold cutoff, and both the *t*-test and Mann-Whitney *U* test values must be < 0.002.

To improve the translatability of the cell line-derived signature to human tumors, we used PCA on human lung adenocarcinomas from several external public datasets to remove genes from the signature that did not translate into human tumors. The datasets used in determining translatability were GEO [44] datasets GSE30219, GSE37745, and the Director's Challenge Plus (Director's Challenge [45] + sister samples from GSE14814). Each dataset was normalized separately with IRON and then de-batched as appropriate with COMBAT [46]. PCA was performed using Evince (Prediktera, Umeå, Sweden). After removing those genes that did not translate, the loadings from the first two principal components were used to visualize sign agreement between the original cell line-derived signature and the signature behavior in each dataset, resulting in the final 105-probeset *TGFβ-EMT* signature (representing 77 genes).

### NanoString analysis

Due to lack of codeset availability or low agreement between FFPE and fresh frozen assays, the resulting 77 genes were further reduced to a set of 60 genes for use with the MFS NanoString (NanoString Technologies, Seattle, WA) analyses. NanoString assays were performed on RNA extracted from FFPE blocks corresponding to a 136-sample subset of a 442 human adenocarcinoma dataset (Schabath 442) [28].

### Kaplan–Meier analysis

OS and MFS were estimated using Kaplan-Meier analysis, applied to three sample cohorts: a 136 sample subset (three of the 136 NanoString samples had pre-existing metastases at time of surgery, and were thus excluded from MFS analysis) of the Schabath 442 fresh frozen tumor microarray dataset [45], NanoString assays of RNA isolated from FFPE blocks of these same 136 tumors, and a combined cohort of the two datasets from Nguyen *et al.* [29]. For MFS analysis, metastasis and death were both counted as events. High and low *TGFβ-EMT* signature groupings were determined by applying the 105-probeset signature to each dataset using PCA, and then using the median of the first principal component as the cutoff point. *P-*values were calculated using the log-rank test.

### Colon metastatic-potential cohort

The *TGFβ-EMT* signature was applied to a cohort of 96 colon tumors from patients metastasis-free at diagnosis [47] that were normalized with IRON. The first principal component was used to assign *TGFβ-EMT* signature strength to each tumor, and a two-group comparison between patients that had metastasized at the time of tumor resection and those that had not was performed using a two-sided *t*-test.

### Immunohistochemical staining and evaluation

A previously described lung adenocarcinoma Tissue Microarray (TMA) was used for these studies [34]. This TMA included 150 cores from primary adenocarcinomas, 58 cores of adjacent normal lung tissue, 14 cores from non-lung tissue controls (normal and cancer), and 10 samples of lung cancer cell lines. TMA slides were cut into 4-μm sections and stained in the Moffitt Pathology Core with antibodies against CDH1 (#760-4440, Cell Marque, Rocklin, CA), SMAD7 (ab76498, Abcam, Cambridge, MA), PLAUR (PA5-15478, Thermo Fisher Scientific), A2A (ab3461, Abcam; BTLA, LS-B9823, Lifespan Bioscience, Seattle, WA), CTLA4 (sc-376016, Santa Cruz), INOS (ab3523, Abcam; Ki67, 790-4286, Ventana, Tucson, AZ), LAG3 (ab180187, Abcam), PDL1 (#13684, Cell Signaling, Danvers, MA), and TIM3 (AF2365, R&D Systems). Antigen retrieval and incubation times were optimized for each antibody as follows: 60-minute retrieval for CTLA4, TIM3, and SMAD7 antibodies, and 32-minute incubation for the A2A, BTLA, LAG3, and INOS antibodies. The Ventana Benchmark XT platform was used for all immunohistochemistry analyses. Each TMA slide was scanned using the Aperio (Vista, CA) ScanScope XT with a ×20/0.8 numerical aperture objective lens at a rate of 10 minutes per slide. Image analyses for stained TMAs were performed using Aperio Nuclear v9.1 to segment nuclei of various intensities.

The data discussed in this publication have been deposited in NCBI's Gene Expression Omnibus (Edgar *et al*., 2002) and are accessible through GEO Series accession number GSE114761 (https://www.ncbi.nlm.nih.gov/geo/query/acc.cgi?acc=GSE114761).

## SUPPLEMENTARY MATERIALS FIGURES AND TABLES





## Abbreviations

*ANGPTL4*: Angiopoietin-like 4
BATTLE-1: Biomarker-integrated Approaches of Targeted Therapy for Lung Cancer Elimination
CDH1: E-Cadherin 1
EMT: Epithelial-to-Mesenchymal Transition
FFPE: Formalin-Fixed Paraffin Embedded
MFS: Metastasis Free Survival
NSCLC: Non-Small Cell Lung Carcinoma
OS: Overall Survival
PCA: Principal Component Analysis
PROSPECT: Profiling of Resistance patterns and Oncogenic Signaling Pathways in Evaluation of Cancers of the Thorax
TCGA: The Cancer Genome Atlas
TGFβ: Transforming Growth Factor beta (b)
*TGFβ-EMT* signature: 77 genes expression signature associated with TGFβ-induced EMT activities
*TGFβ-EMT_N_* signature: NanoString signature utilizing a subset of 60 of the 77 genes in the TGFβ-EMT signature
TMA: Tissue Microarray
