# Correction: Transforming growth factor β-induced epithelial-to-mesenchymal signature predicts metastasis-free survival in non-small cell lung cancer

**DOI:** 10.18632/oncotarget.28004

**Published:** 2021-06-22

**Authors:** Edna Gordian, Eric A. Welsh, Nicholas Gimbrone, Erin M. Siegel, David Shibata, Ben C. Creelan, William Douglas Cress, Steven A. Eschrich, Eric B. Haura, Teresita Muñoz-Antonia

**Affiliations:** ^1^ Tumor Biology Program, H. Lee Moffitt Cancer Center and Research Institute, Tampa, FL, USA; ^2^ Cancer Informatics Core, H. Lee Moffitt Cancer Center and Research Institute, Tampa, FL, USA; ^3^ Molecular Oncology Program, H. Lee Moffitt Cancer Center and Research Institute, Tampa, FL, USA; ^4^ Cancer Epidemiology Program, H. Lee Moffitt Cancer Center and Research Institute, Tampa, FL, USA; ^5^ Department of Surgery, University of Tennessee Health Science Center, Memphis, TN, USA; ^6^ Department of Thoracic Oncology, H. Lee Moffitt Cancer Center and Research Institute, Tampa, FL, USA; ^7^ Department of Biostatistics and Bioinformatics, H. Lee Moffitt Cancer Center and Research Institute, Tampa, FL, USA; ^*^ These authors contributed equally to this work

**This article has been corrected:** The ‘Antibodies and Compounds’ section has been updated to read, “The following antibodies were purchased from Cell Signaling Technology (Danvers, MA): anti-Smad2 and loading control antibodies (antiGAPDH or anti-beta actin).” In addition, the ‘Quantitative reverse transcription-PCR (qRT-PCR)’ section has been updated to read, “Primers for SERPINE, SMAD7, SNAI1, PLAUR, and MUC5AC were purchased….” The authors declare that these corrections do not change the results or conclusions of this paper.

Original article: Oncotarget. 2019; 10:810–824. 810-824. https://doi.org/10.18632/oncotarget.26574

