# Supplementary material for: Transforming growth factor β-induced epithelial-to-mesenchymal signature predicts metastasis-free survival in non-small cell lung cancer
Source: Oncotarget. 2019 Jan 25;10(8):810–24. doi: 10.18632/oncotarget.26574 (PMC6368226; doi:10.18632/oncotarget.26574)
Supplement: Supplementary file 1 [file oncotarget-10-810-s001.pdf]

Transforming growth factor β-induced epithelial-to-mesenchymal signature predicts metastasis-free survival in non-small cell lung cancer

SUPPLEMENTARY MATERIALS

Antibodies and compounds

The following antibodies were purchased from Cell Signaling Technology (Danvers, MA): anti-pSma2 and anti-GAPDH.

Quantitative reverse transcription-PCR (qRT-PCR)

RNA was extracted from untreated and TGFβ treated cells with TRIZOL Reagent (Invitrogen) and was reversed transcribed using High Capacity cDNA Transcription Kit (Life Technologies). Primers for *SERPINE, SMAD7, SNAI1, SMURF1, and RUNX1* were purchased from Applied Biosystems. Quantitative RT-PCR reactions were set up in triplicates using the corresponding Taqman Gene Expression Assay from Life technologies, and carried out on a 7900HT Fast Real-Time PCR System (Applied Biosystems). mRNA levels were determined by the comparative Ct method, normalizing untreated and treated samples to an internal control (GAPDH) and expression relative to the untreated sample.

Proliferation/Cytotoxicity

A549 cells were seeded in 96-well plates at 5 × 10^3 cells per well in triplicate. After 24 hours, cells were treated with the indicated concentrations of TGFβ-1. After a 48-hour incubation, proliferation/cytotoxicity was measured by adding CellTiter 96 One Solution (Promega Corporation, Madison, WI). After addition, cells were left to incubate for 1 hour, and absorbance was measured at 490 nm in a BioTeK EL808 microplate reader (BioTek Instruments Inc, Winooski, VT). Treated samples were normalized to untreated controls with results shown as percentages.

Plasmids and liposome-mediated gene transfer

The p3TP-Lux luciferase reporter contains three repeats of the TPA-responsive element (TRE) fused to a portion of the PAI-1 promoter (provided by J. Massagué, Sloan Kettering Cancer Center, New York). Transient transfections were performed as described previously (Muñoz-Antonia, T., Li, X., Reiss, M., Jackson, R., and Antonia, S. (1996) *Cancer Res.* 56, 4831-5) with some modifications. Briefly, plasmid DNA (1.7 µg of the luciferase reporter plasmid) were mixed with 10 µL of Fugene reagent (Roche Diagnostic) and incubated for 15 minutes at room temperature before addition to semiconfluent cell cultures in 60 mm tissue culture dishes. Four hours after the start of transfection, cells were treated with 5 ng/ml TGFb-1. Forty-eight hours after the start of the transfection, the amount of luciferase enzyme activity in cell extracts was determined using the Luciferase Assay System (Promega Corporation). Ten µL of the cell extracts were added to 50 µL of the Luciferase reagent and the amount of light produced was measured using a Barthold Luminometer (Wallac, Inc., Gaithersburg, MD). The amount of protein present in the cell extracts was determined using the Bio-Rad Bradford assay.

Western blotting

Whole-cell protein extraction was performed by scrapping the cells in cold 1× phosphate-buffered saline (PBS), followed by sonication and lysis in 1× CHAPS buffer (Cell Signaling Technology). Protein concentrations were determined using the Bradford Assay (Bio-Rad, Hercules, CA). Protein lysates were resolved by sodium dodecyl sulfate–polyacrylamide gel electrophoresis (SDS-PAGE), transferred to a polyvinylidene fluoride membrane (Millipore Corporation, Billerica, MA), blocked for 1 hour in 1× TBST containing 5% nonfat milk, and incubated overnight in corresponding primary antibody at 4° C. Blots were finished by incubation with horseradish peroxidase-labeled secondary antibody and developed using Amersham ECL Prime Western Blotting Detection Reagent (GE Healthcare Life Sciences, Pittsburg, PA).

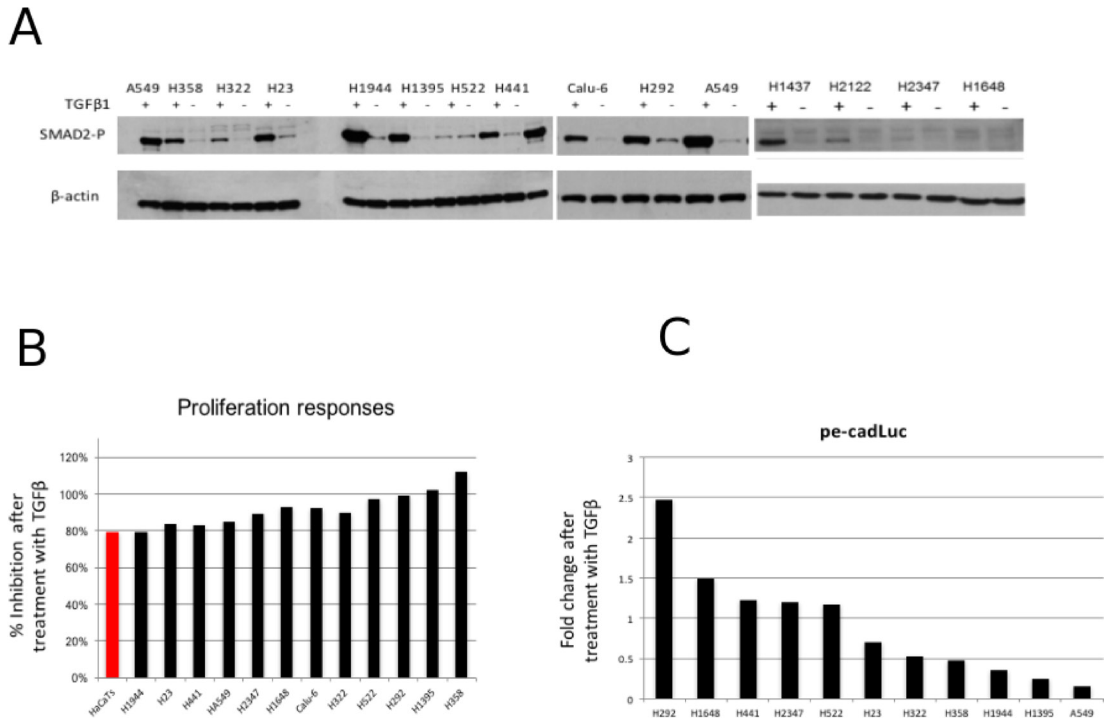

Supplementary Figure 1: Characterization of NSCLC cell lines based on TGFβ responses and EMT capacity. (A) NSCLC cell lines were treated with 5 ng/mL TGFβ-1 for 48 hours. After incubation, cells were harvested, lysed, and Smad2 phosphorylation detected by Western Blot analysis. (**B**) NSCLC cells were treated with 5 ng/ml TGFβ-1 for 48-hours, and proliferation/cytotoxicity was measured by adding CellTiter 96 One Solution. Data generated by cell viability assay are expressed as a percentage of the value for untreated cells. A normal epithelial cell line (HaCaT) was used as a control for TGFβ-1 inhibition. **(C)** TGFβ-1-dependent transcriptional responses were determined with a luciferase assay using p3TPLuc, a plasmid that has three TGFβ responsive elements and should only show activity after TGFβ treatment. Transcriptional response to p3TPLux was proportional to the mesenchymal status of each cell line.

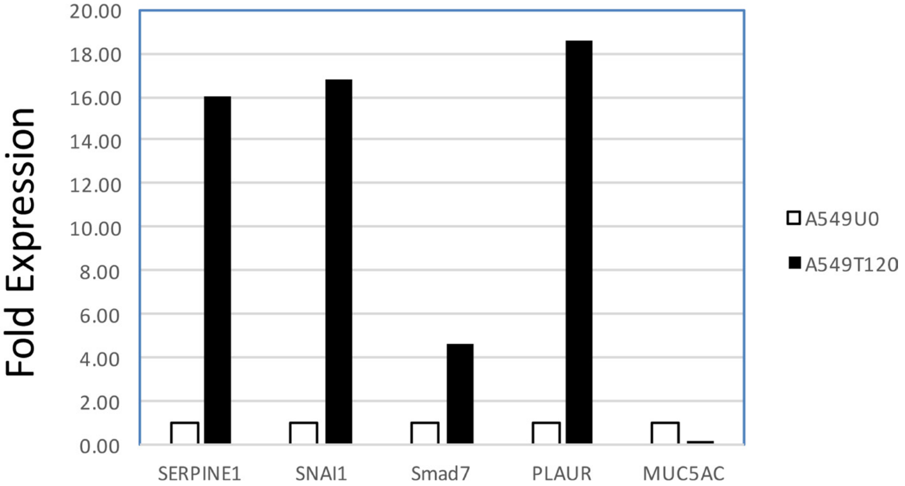

Supplementary Figure 2: Validation of microarray changes in gene expression after TGFβ-1 treatment using quantitative real-time RT-PCR. The mRNA levels of TGFβ-1 responsive genes in A549 cells were measured by quantitative real-time RT-PCR after treatment with TGFβ-1 for 120 hours. Values are expressed by fold change between time 0 (A549 U0) and 120 hours (A549T120). Determinations were done in triplicate.

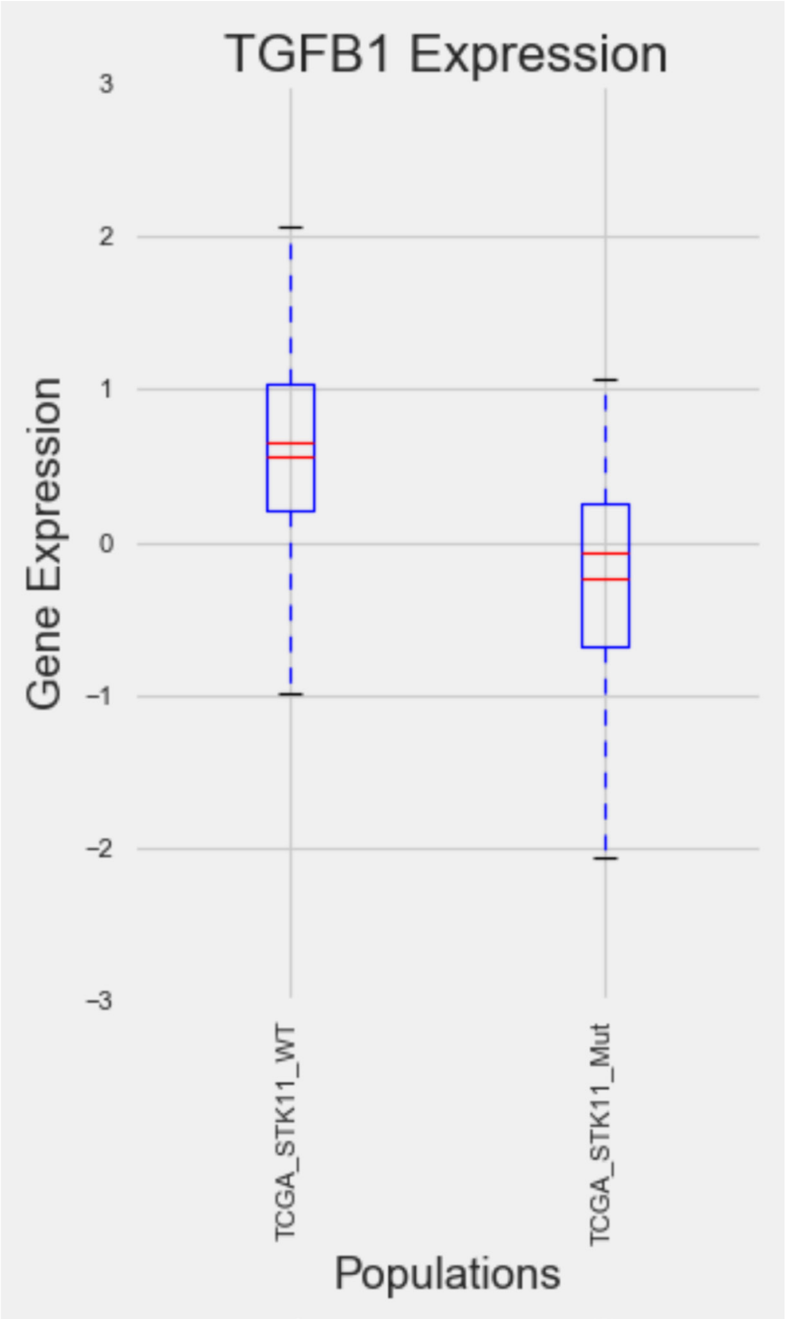

Supplementary Figure 3: TGFβ-1 expression in TCGA patients with and without STK11 mutations.

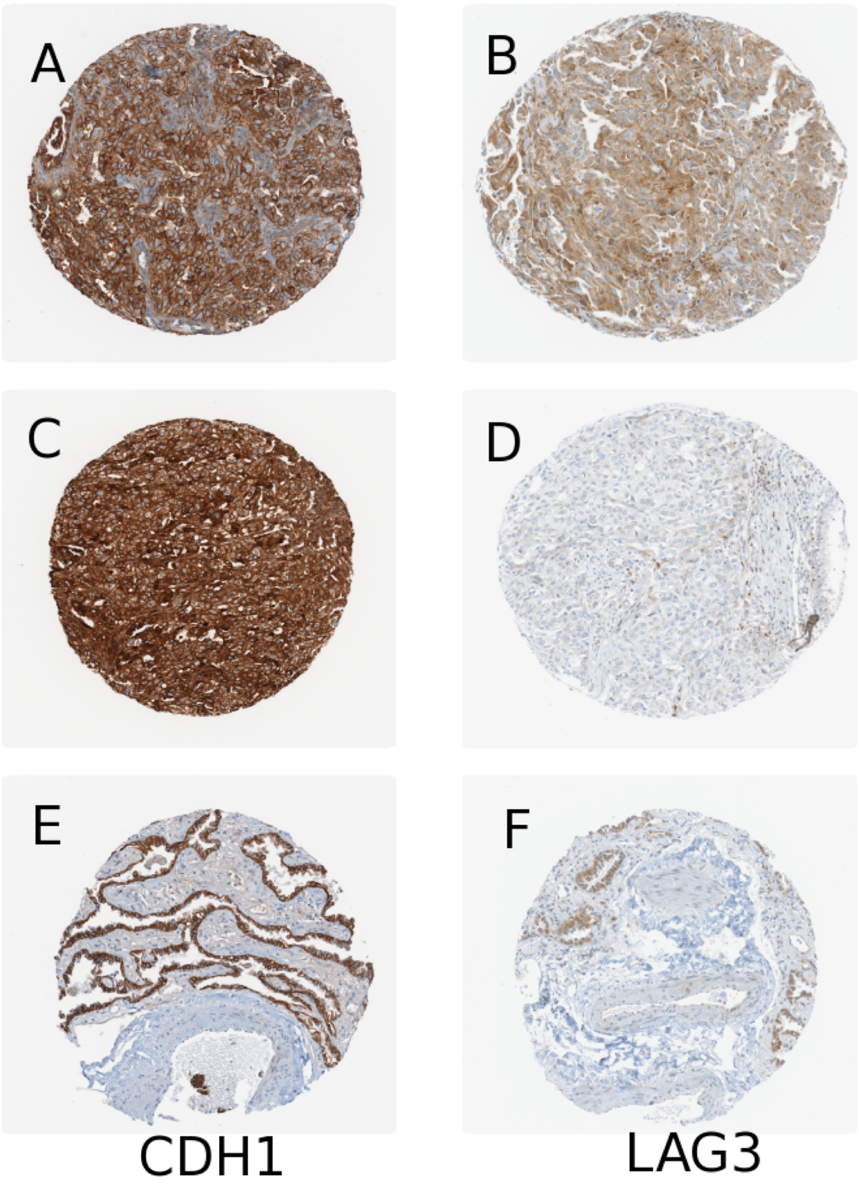

Supplementary Figure 4: Immunohistochemical detection of E-Cadherin (CDH1) and LaG3 proteins in NSCLC patients included in the lung adenocarcinoma TMA. Representative CDH1 expression in tumors from lung adenocarcinoma patients without metastasis (**A**/**B** and **E**/**F**); and in a patient with metastasis (**C**/**D**). Even if CDH1 expression is very strong, the patient in (**C**) (Core #61) has high *TGFβ-EMT* (0.82) and $TGF\beta\text{-}EMT_N$ (0.70) signatures scores, suggesting metastatic potential. Supplementary Table 2 has the details of the OS, MFS, Aperio immunohistochemistry results and *TGFβ-EMT* or $TGF\beta\text{-}EMT_N$ signatures scores for all the samples included in the NSCLC TMA.

Supplementary Table 1: Genes included in the *TGFβ-EMT*$_N$ signature

Symbol		
ACTN1	*GADD45B*	*PMEPA1*
ADGRF4	*GALNT2*	*PTRF*
AFAP1L2	*ITGA5*	*RHOD*
ANKLE2	*JUNB*	*SAMD4A*
ARHGEF18	*KLF7*	*SERPINE1*
ARHGEF40	*LAMC2*	*SKIL*
BEAN1	*LTBP1*	*SMAD7*
BICDL1*[*]	*LTBP3*	*SNAI1*
BMP1	*LTBP4*	***TBC1D30****[*]
CALD1	*MAF*	*TGFB1I1*
CERCAM	***MAPRE2****[*]	*TGFBR1*
CHST3	*MBOAT2*	***THRB****[*]
CMTM3	*MLXIP*	*TIMP2*
COL1A1	***MUC5AC****[*]	***TMC5****[*]
COL7A1	***MUC5B****[*]	*TP53I3*
DBN1	*NAV1*	*TPM1*
DEPTOR*[*]	*NCOR2*	*TRIO*
EPB41L5	*PDLIM7*	*TSPAN2*
EXOC6*[*]	*PEA15*	*VCAN*
FRMD6	*PLEK2*	

[*]= Negative PCA coefficient.

Supplementary Table 2: Overall survival (OS), Metastasis Free Survival (MFS), Aperio E-cadherin and Lag-3 immunohistochemistry results (Frac Positive) and *TGFβ-EMT* or *TGFβ-EMT*$_N$ signatures scores for all the samples included in the NSCLC TMA. See Supplementary_Table_2
