# Supplementary material for: Transforming growth factor β-induced epithelial-to-mesenchymal signature predicts metastasis-free survival in non-small cell lung cancer
Source: Oncotarget. 2019 Jan 25;10(8):810–24. doi: 10.18632/oncotarget.26574 (PMC6368226; doi:10.18632/oncotarget.26574)
Supplement: Supplementary file 2 [file oncotarget-10-810-s002.docx]

**Supplementary Table 2: Overall survival (OS), Metastasis Free Survival (MFS), Aperio E-cadherin and Lag-3 immunohistochemistry results (Frac Positive) and *TGFβ-EMT* or *TGFβ-EMT_N_* signatures scores for all the samples included in the NSCLC TMA**

| **Col** | **Row** | **OS_ months** | **OS_ event** | **MFS_ months** | **MFS_ event** | *TGFβ-EMT* **t[1] Microarray** | *TGFβ-EMT_N_* **t[1] NanoString** | **CDH1 FracPos** | **LAG3 FracPos** |
| --- | --- | --- | --- | --- | --- | --- | --- | --- | --- |
| A | 1 | 33.74 | Event | 19.52 | Event | 2.53 | 4.29 | 0.72 | 0.20 |
| A | 7 | 57.07 | Event | 37.29 | Event | 0.48 | 1.22 | 0.98 | 0.08 |
| A | 8 | 1.91 | Event | 1.91 | Event | -6.82 | 9.07 | 0.93 | 0.01 |
| A | 9 | 11.40 | Event | 11.40 | Event | 7.47 | 2.74 | 0.81 | 0.27 |
| A | 10 | 9.30 | Event | 9.30 | Event | -4.89 | 3.71 | 0.76 | 0.12 |
| A | 12 | 43.27 | Censor | 43.27 | Censor | -1.22 | 2.69 | 0.93 | 0.00 |
| A | 13 | 3.71 | Event | 1.45 | Event | 1.82 | -5.21 | 0.51 | 0.06 |
| A | 14 | 42.12 | Censor | 42.12 | Censor | 2.00 | 0.24 | 0.38 | 0.19 |
| A | 16 | 33.31 | Event | 4.90 | Event | 10.64 | 3.27 | 0.87 | 0.04 |
| A | 18 | 41.89 | Censor | 41.89 | Censor | 5.84 | 3.53 | 0.72 | 0.03 |
| A | 19 | 44.58 | Censor | 44.58 | Censor | -3.86 | -3.75 | 0.78 | 0.37 |
| B | 3 | 28.32 | Event | 4.37 | Event | 10.27 | 1.65 | 0.90 | 0.43 |
| B | 4 | 4.30 | Event | NA | Exclude | 2.66 | 2.96 | 0.04 | 0.10 |
| B | 6 | 72.02 | Censor | 72.02 | Censor | -13.00 | -9.30 | 0.98 | 0.00 |
| B | 8 | 54.97 | Censor | 54.97 | Censor | 8.85 | 9.21 | 0.48 | 0.25 |
| B | 10 | 43.73 | Censor | 43.73 | Censor | -0.57 | -3.37 | 0.04 | 0.02 |
| B | 11 | 66.69 | Censor | 66.69 | Censor | -6.22 | 1.42 | 0.82 | 0.06 |
| B | 12 | 13.63 | Event | 8.21 | Event | -0.42 | -0.33 | 0.94 | 0.00 |
| B | 14 | 60.02 | Censor | 60.02 | Censor | -1.96 | 1.38 | 0.52 | 0.22 |
| B | 15 | 24.05 | Event | 24.05 | Event | 0.43 | 2.23 | 0.68 | 0.16 |
| B | 16 | 0.76 | Event | 0.76 | Event | 4.51 | 2.86 | 0.46 | 0.19 |
| B | 17 | 50.83 | Censor | 50.83 | Censor | -2.57 | -2.11 | 0.51 | 0.19 |
| B | 19 | 4.67 | Censor | 4.67 | Censor | -2.61 | 11.10 | 0.81 | 0.08 |
| B | 20 | 17.77 | Event | 17.77 | Event | -11.48 | -8.02 | 0.94 | 0.02 |
| C | 1 | 2.63 | Event | 2.63 | Event | 2.47 | 3.31 | 0.67 | 0.02 |
| C | 3 | 2.40 | Event | 2.40 | Event | 5.14 | 4.58 | 0.37 | 0.14 |
| C | 4 | 64.16 | Censor | 42.68 | Event | -3.02 | -4.14 | 0.48 | 0.11 |
| C | 5 | 64.56 | Event | 64.56 | Event | 1.20 | -0.80 | 0.67 | 0.27 |
| C | 7 | 10.78 | Event | 8.02 | Event | 5.14 | 0.76 | 0.84 | 0.04 |
| C | 9 | 68.27 | Event | 15.54 | Event | 9.11 | 5.82 | 0.94 | 0.09 |
| C | 10 | 27.47 | Event | 27.47 | Event | 8.72 | 10.04 | 0.94 | 0.23 |
| C | 11 | 7.10 | Event | 7.10 | Event | 7.97 | 3.27 | 0.98 | 0.02 |
| C | 12 | 54.37 | Censor | 54.37 | Censor | 2.96 | 0.39 | 0.55 | 0.01 |
| C | 14 | 8.77 | Event | 6.18 | Event | 15.80 | 3.83 | 0.81 | 0.20 |
| C | 15 | 13.08 | Event | 13.08 | Event | -0.06 | -1.02 | 0.71 | Empty |
| C | 17 | 38.51 | Censor | 38.51 | Censor | 0.32 | -2.94 | 0.93 | 0.01 |
| C | 19 | 30.75 | Event | 16.39 | Event | 4.13 | 7.78 | 0.72 | 0.17 |
| D | 2 | 45.08 | Event | 45.08 | Event | -3.33 | -4.89 | 0.97 | 0.03 |
| D | 4 | 14.13 | Event | 8.94 | Event | 13.44 | 3.30 | 0.80 | 0.11 |
| D | 5 | 16.07 | Event | 5.91 | Event | 2.66 | 0.92 | 0.91 | 0.26 |
| D | 6 | 34.07 | Event | 34.07 | Event | -8.47 | -7.47 | 0.82 | 0.63 |
| D | 8 | 46.65 | Censor | 46.65 | Censor | -2.55 | -5.41 | 0.91 | 0.27 |
| D | 11 | 18.30 | Censor | 18.30 | Censor | 1.39 | 1.61 | 0.97 | 0.23 |
| D | 12 | 7.10 | Event | NA | Exclude | 3.12 | -0.88 | 0.98 | 0.23 |
| D | 13 | 8.80 | Censor | 8.80 | Censor | -7.83 | -0.53 | 0.55 | 0.36 |
| D | 14 | 6.18 | Event | 0.82 | Event | 1.01 | -2.90 | 0.88 | 0.20 |
| D | 15 | 32.76 | Censor | 32.76 | Censor | -1.22 | 1.02 | 0.98 | 0.01 |
| D | 17 | 34.96 | Censor | 34.96 | Censor | -4.41 | 2.56 | 0.02 | 0.06 |
| D | 18 | 10.61 | Event | 10.61 | Event | 6.40 | 1.36 | 0.29 | 0.17 |
| D | 19 | 59.14 | Censor | 33.64 | Event | -3.83 | 0.03 | 0.99 | 0.05 |
| D | 20 | 46.29 | Censor | 11.66 | Event | 5.53 | 7.68 | 0.98 | 0.05 |
| E | 1 | 57.10 | Censor | 57.10 | Censor | 3.30 | 2.21 | 0.72 | 0.17 |
| E | 4 | 64.72 | Event | 64.26 | Event | -5.19 | -0.82 | 0.48 | 0.32 |
| E | 7 | 59.47 | Censor | 59.47 | Censor | 6.28 | -0.50 | 0.81 | 0.14 |
| E | 8 | 70.97 | Censor | 70.97 | Censor | -3.47 | -3.76 | 0.47 | 0.17 |
| E | 9 | 6.34 | Event | 6.34 | Event | -9.16 | -3.14 | 0.70 | 0.15 |
| E | 10 | 55.62 | Censor | 55.62 | Censor | 1.92 | 3.29 | 0.98 | 0.26 |
| E | 11 | 68.76 | Censor | 68.76 | Censor | 5.73 | -4.37 | 0.24 | 0.19 |
| E | 14 | 37.72 | Censor | 37.72 | Censor | -7.18 | -0.25 | 0.67 | 0.56 |
| E | 18 | 26.58 | Event | 26.58 | Event | 3.06 | 5.11 | 0.76 | 0.08 |
| E | 19 | 53.19 | Censor | 53.19 | Censor | -4.83 | -6.69 | 0.85 | 0.21 |
| F | 1 | 52.93 | Censor | 52.93 | Censor | 6.82 | -2.40 | 0.70 | 0.31 |
| F | 2 | 64.39 | Censor | 64.39 | Censor | 1.40 | 1.65 | 0.38 | 0.16 |
| F | 5 | 43.17 | Event | 40.21 | Event | 9.69 | 3.37 | 0.90 | 0.05 |
| F | 6 | 32.30 | Event | 20.50 | Event | 2.25 | -4.02 | 0.49 | Empty |
| F | 7 | 67.91 | Censor | 7.00 | Event | -4.72 | 3.03 | 0.98 | 0.06 |
| F | 10 | 28.09 | Censor | 28.09 | Censor | 4.13 | 0.67 | 0.49 | 0.19 |
| F | 12 | 7.85 | Event | 7.85 | Event | 2.55 | -0.79 | 0.79 | 0.08 |
| F | 14 | 34.79 | Censor | 34.79 | Censor | -8.05 | -4.58 | 0.69 | 0.22 |
| F | 16 | 86.41 | Censor | 86.41 | Censor | -4.83 | -1.91 | 0.52 | 0.42 |
| F | 17 | 7.69 | Event | 7.69 | Event | 1.15 | 0.33 | 0.60 | 0.04 |
| G | 2 | 33.08 | Event | 13.24 | Event | 3.27 | 4.50 | 0.56 | 0.15 |
| G | 4 | 55.82 | Event | 55.82 | Event | -10.73 | -9.38 | 0.95 | 0.35 |
| G | 6 | 22.34 | Event | 22.34 | Event | 8.95 | 1.28 | 0.40 | 0.16 |
| G | 7 | 20.30 | Event | 20.30 | Event | 10.85 | 6.16 | 0.82 | 0.70 |
| G | 8 | 50.89 | Censor | 50.89 | Censor | -7.05 | -6.80 | 0.36 | 0.46 |
| G | 10 | 75.50 | Event | 59.79 | Event | 1.63 | -4.06 | 0.26 | 0.06 |
| G | 11 | 56.18 | Censor | 56.18 | Censor | 3.41 | 1.01 | 0.94 | 0.36 |
| G | 13 | 65.25 | Censor | 65.25 | Censor | 3.27 | 5.42 | 0.50 | 0.42 |
| G | 15 | 45.67 | Event | 45.67 | Event | 1.76 | -1.58 | 0.14 | 0.07 |
| G | 16 | 20.60 | Event | 14.72 | Event | -11.78 | -1.53 | 0.99 | 0.02 |
| G | 17 | 26.74 | Event | 26.74 | Event | -0.59 | 1.01 | 0.67 | 0.31 |
| G | 19 | 22.05 | Censor | 22.05 | Censor | 7.23 | -4.28 | 0.77 | 0.37 |
| H | 2 | 38.67 | Censor | 38.67 | Censor | 7.44 | -4.45 | 0.89 | 0.00 |
| H | 6 | 58.87 | Censor | 58.87 | Censor | -1.11 | -2.67 | 0.86 | 0.47 |
| H | 8 | 90.02 | Censor | 62.85 | Event | -4.28 | 1.39 | 0.81 | 0.09 |
| H | 9 | 5.62 | Event | 5.62 | Event | 4.99 | 5.88 | 0.93 | 0.05 |
| H | 11 | 50.73 | Event | 50.73 | Event | 1.04 | 0.89 | 0.41 | 0.18 |
| H | 12 | 49.54 | Censor | 49.54 | Censor | -3.09 | -1.63 | 0.98 | 0.10 |
| H | 13 | 58.22 | Event | 58.22 | Event | -5.44 | -3.27 | 0.32 | 0.22 |
| H | 14 | 74.02 | Censor | 74.02 | Censor | -3.35 | -6.39 | 0.89 | 0.29 |
| H | 15 | 31.24 | Event | 31.24 | Event | 9.93 | 1.70 | 0.60 | Empty |
| H | 17 | 38.18 | Event | 38.18 | Event | 5.71 | 0.01 | 0.82 | Empty |
| H | 18 | 14.52 | Event | 14.52 | Event | 3.23 | 0.47 | 0.98 | 0.06 |
| H | 19 | 15.64 | Event | 15.64 | Event | 4.53 | 8.56 | 0.74 | 0.11 |
| I | 1 | 72.41 | Censor | 72.41 | Censor | 4.78 | 1.41 | 0.40 | 0.10 |
| I | 2 | 42.38 | Censor | 42.38 | Censor | -11.76 | -5.71 | 0.61 | 0.05 |
| I | 3 | 67.15 | Censor | 67.15 | Censor | 0.14 | -0.29 | 0.92 | 0.25 |
| I | 5 | 62.23 | Censor | 62.23 | Censor | -10.13 | -2.91 | 0.28 | 0.07 |
| I | 7 | 82.83 | Censor | 82.83 | Censor | -10.64 | -8.69 | 0.62 | 0.27 |
| I | 9 | 35.29 | Event | 16.43 | Event | -15.99 | -1.74 | 0.67 | 0.17 |
| I | 10 | 58.09 | Censor | 17.28 | Event | 6.26 | 4.96 | 0.30 | 0.08 |
| I | 12 | 66.23 | Censor | 66.23 | Censor | -0.87 | -4.12 | 0.06 | 0.24 |
| I | 15 | 39.98 | Event | 39.98 | Event | -4.59 | -5.58 | 0.89 | 0.24 |
| I | 17 | 40.74 | Censor | 40.74 | Censor | -5.05 | -4.42 | 0.58 | 0.07 |
| I | 18 | 83.06 | Censor | 83.06 | Censor | -3.43 | -9.53 | 0.74 | 0.02 |
| I | 20 | 48.03 | Censor | 48.03 | Censor | -5.20 | -3.42 | 0.78 | 0.16 |
| J | 3 | 44.48 | Event | 44.48 | Event | 1.93 | 0.58 | 0.93 | 0.17 |
| J | 5 | 32.36 | Event | 32.36 | Event | -0.39 | -0.62 | 0.85 | 0.64 |
| J | 6 | 16.69 | Event | 16.69 | Event | 9.65 | 5.58 | 0.61 | 0.07 |
| J | 8 | 29.96 | Event | 21.85 | Event | -21.27 | -16.94 | 0.91 | 0.11 |
| J | 9 | 49.18 | Censor | 49.18 | Censor | -7.44 | -4.10 | 0.92 | 0.52 |
| J | 11 | 7.33 | Event | 7.33 | Event | 3.53 | 4.63 | 0.56 | Empty |
| J | 14 | 66.69 | Censor | 66.69 | Censor | -6.86 | -1.06 | 0.28 | 0.06 |
| J | 15 | 14.32 | Event | 14.32 | Event | -0.75 | 5.22 | 0.99 | 0.01 |
| J | 17 | 36.44 | Censor | 36.44 | Censor | -10.66 | -8.79 | 0.72 | 0.62 |
| J | 18 | 61.40 | Censor | 61.40 | Censor | -5.34 | 2.72 | 0.65 | Empty |
| K | 6 | 11.76 | Event | 5.42 | Event | 6.56 | 3.62 | 0.94 | 0.09 |
| K | 8 | 27.73 | Event | NA | Exclude | -5.09 | -2.73 | 0.99 | 0.27 |
| K | 10 | 7.20 | Event | 3.45 | Event | -2.46 | -3.07 | 0.82 | 0.09 |
| K | 11 | 9.63 | Event | 3.81 | Event | 2.80 | 5.88 | 0.99 | 0.00 |
| K | 13 | 79.38 | Censor | 33.12 | Event | -2.00 | -4.24 | 0.71 | 0.06 |
| K | 15 | 71.75 | Censor | 71.75 | Censor | 6.49 | 4.78 | 0.24 | Empty |
| K | 16 | 47.47 | Censor | 47.47 | Censor | 0.29 | 1.32 | 0.64 | 0.01 |
| K | 17 | 59.14 | Censor | 16.79 | Event | -7.35 | 0.05 | 0.40 | 0.22 |
| K | 18 | 35.75 | Censor | 35.75 | Censor | 1.67 | 0.19 | 0.82 | 0.23 |
| K | 20 | 58.18 | Censor | 58.18 | Censor | 0.16 | 5.11 | 0.56 | 0.24 |
| L | 4 | 16.07 | Event | 16.07 | Event | -3.49 | 3.93 | 0.37 | 0.22 |
| L | 5 | 45.27 | Censor | 45.27 | Censor | -1.25 | -1.73 | 0.31 | 0.14 |
| L | 7 | 63.21 | Censor | 63.21 | Censor | -0.31 | 2.88 | 0.96 | 0.01 |
| L | 9 | 92.16 | Censor | 92.16 | Censor | -3.70 | -2.04 | 0.11 | 0.10 |
| L | 11 | 0.49 | Event | 0.49 | Event | 9.19 | -0.97 | 0.33 | 0.17 |
| L | 13 | 22.37 | Event | 22.37 | Event | -0.28 | 1.48 | 0.56 | 0.60 |
| L | 17 | 50.04 | Censor | 50.04 | Censor | -6.27 | -0.82 | 0.68 | Empty |
| L | 19 | 61.63 | Censor | 21.39 | Event | -0.82 | -0.13 | 0.38 | 0.10 |
| L | 20 | 42.58 | Event | 42.58 | Event | 5.61 | 1.82 | 0.91 | 0.02 |
